# Are patients who call a primary care office referred to the emergency department by non-healthcare personnel without the input of a physician?

**DOI:** 10.7717/peerj.1507

**Published:** 2016-03-28

**Authors:** Russell Hill, Albert Gest, Cynthia Smith, Jose H. Guardiola, Michael Apolinario, Joann Ha, Jose R. Gonzalez, Peter B. Richman

**Affiliations:** 1 Department of Emergency Medicine, Christus Spohn/Texas A&M Health Science Center, Corpus Christi, TX, United States; 2 Department of Mathematics, Texas A&M University - Corpus Christi, Corpus Christi, TX, United States; 3 Department of Internal Medicine, University of Texas Health Science Center Northeast-Good Shepherd Medical Center, Longview, TX, United States

**Keywords:** Primary care referral, Emergency department, Utilization

## Abstract

**Objective.** We hypothesized that a significant percentage of patients who are referred to the Emergency Department (ED) after calling their primary care physician’s (PCP) office receive such instructions without the input of a physician.

**Methods.** We enrolled a convenience sample of stable adults at an inner-city ED. Patients provided written answers to structured questions regarding PCP contact prior to the ED visit. Continuous data are presented as means ± standard deviation; categorical data as frequency of occurrence. 95% confidence intervals were calculated.

**Results.** The study group of 660 patients had a mean age of 41.7 ± 14.7 years and 72.6% had income below $20,000/year. 472 patients (71.51%; 67.9%–74.8%) indicated that they had a PCP. A total of 155 patients (23.0%; 19.9%–26.4%) called to contact their PCP prior to ED visit. For patients who called their PCP office and were directed by phone to the ED, the referral pattern was observed as follows: 31/98 (31.63%; 23.2%–41.4%) by a non-health care provider without physician input, 11/98 (11.2%; 6.2%–19.1%) by a non-healthcare provider after consultation with a physician, 12/98 (12.3%; 7.7%–20.3%) by a nurse without physician input, and 14/98 (14.3%; 8.6%–22.7%) by a nurse after consultation with physician. An additional 11/98, 11.2%; 6.2–19.1%) only listened to a recorded message and felt the message was directing them to the ED.

**Conclusion.** A relatively small percentage of patients were referred to the ED without the consultation of a physician in our overall population. However, over half of those that contacted their PCP’s office felt directed to the ED by non-health care staff.

## Introduction

US health care costs and expenditures continue to accelerate with minimal impact by existing dampening measures (National Health Expenditures, 2011; National Hospital Ambulatory Medical Care Survey, 2010) Recent analysis shows that ED utilization and other expensive medical procedures are on the rise, even as costs to the individual and families becomes unsustainable (NPR, Kaiser Family Foundation, Kennedy School of Government, 2002). Injury, illness, and the subsequent cost of health care are often cited as the leading cause of bankruptcy in the US (Himmelstein et al., 2005). A frequent focus of policy makers and analysts aims to balance emergent medical care that occurs in hospitals with preventative measures in an outpatient medical setting.

Emergency departments, ever searching for quality improvement and cost reduction, stand as the gatekeeper and safety net for the United States health care system. Nearly 80 percent of patients who participated in a National Health survey reported that lack of access to other providers was a significant factor behind a visit to the ED (Farrell et al., 2008; Gindi, Cohen & Kirzinger, 2012). While the focus of newly implemented health care laws and regulations aim to improve access to health care through universal insurance coverage, this change alone may do little for cost containment and actual ability for patients to have access to their primary physician (PCP).

How these two vital arms of the health care system act and interact can aid in understanding how to affect meaningful change on the system. Numerous studies have evaluated non-emergent visits to the ED and the potential for handling these visits in the primary care setting (New England Healthcare Institute, 2010; Massachusetts Division of Health Care Finance and Policy, 2004; Richardson & Hwang, 2001; Lowe et al., 1994; Coleman et al., 2001; Raven et al., 2013; Doran et al., 2013; Rocovich & Patel., 2012; American College of Emergency Physicians, 2011; Behara et al., 2005). However, prior investigators have not examined whether or not the primary care office is a source of ED patient referral that occurs without the involvement of medical decision makers (e.g., office nurse or physician). We conducted a cross sectional study to test the hypothesis that a significant percentage of patients who were referred to the ED after calling their PCPs office would receive such instructions without the input of a physician.

## Methods

### Study design

This was a cross-sectional study designed to evaluate the ED referral process when patients call their PCP office.

### Setting

The study was conducted at Texas A& M/Christus Spohn Memorial Hospital which is a level-two trauma center and serves an inner-city population. The annual Emergency Department (ED) census is 45,000 patients. The Christus Spohn Institutional Review Board approved the reviewed study protocol and determined that it was eligible for exempt status prior to the initiation of data collection.

### Population

Our study included a convenience sample of medically stable, consenting, adult patients age >18 years that presented to the emergency department over a 4-month period with a goal of enrolling a minimum of 600 patients. Trained college students served as the primary enrollers for the study. Patients were excluded for any of the following reasons: refusal to provide consent, pregnancy, and inability to complete the questionnaire due to clinical instability, severe pain, or disorientation as determined by a study physician. Patients who were initially unstable then stabilized and/or had their pain adequately controlled were eligible for enrollment. Patients who were not English or Spanish speaking were also excluded as our survey was only available in those languages.

### Study protocol

Consenting eligible patients were enrolled during a 6-week period (November/December 2013) during hours at which trained research associates were available to assist with data collection. Patients were asked to provide written answers for basic demographic information including sex, age, race, income, and education as well as answers to the primary study questions (Appendix S1). They were subsequently asked six structured questions regarding the circumstances of their illness and decision making, including: the symptoms that brought them into the ED (question 1), and whether he/she called his/her primary care physician or regular doctor that day (question 2). If the answer was either no or “I do not have a doctor,” then the information collected for that patient was considered complete. However, for those patients that responded responded yes for question 2, they were subsequently asked to provide answers to an additional four questions: specifying to whom the patient spoke when they called their primary care physician’s office (question 3), and if that person then spoke with their doctor (question 4). The patient was then asked what the person they spoke with asked them to do (question 5), and finally if an appointment was scheduled, and if so, when (question 6). As the structured questions are objective (i.e., questions of action or events), we did not conduct a validation phase for the data collection instrument.

### Statistical analysis

Data was entered into Excel for Windows (Microsoft Corporation, Redmund, WA, USA) and transported into R Core Team-2013 statistical software (Vienna, Austria) for data analysis. Continuous data are presented as means ± standard deviation. Categorical data are presented as frequency of occurrence and were analyzed by chi-square; alpha was set at 0.05. 95% confidence intervals for proportions were calculated. The primary outcome parameter was the percentage of patients who called their PCP office and were referred to the ED without apparent input from a physician. Secondary outcome parameters included an evaluation of the personnel types that referred patients as well as evaluating for associations between the primary outcome parameter and various patient characteristics/demographics.

## Results

A total of 660 patients were enrolled into the study. Basic demographic characteristics are reviewed in Table 1. The study group was young, poorly educated, and relatively poor. Within the study group, 472 patients (71.51%; 67.9%–74.8%) indicated that they had a PCP. Table 2 summarizes the characteristics of the 155 patients that called their primary care physician’s office before coming to the ED. Of those that called their PCP prior to arriving, we did not find significant differences between patients of different genders, income, and education levels respectively.

**Table 1:** PCP Office Referral Pattern.

Characteristic	
Mean Age	41.7 ± 14.7 years
% female	53.8%
% less than high school education	30.0%
% less than $20,000 annual income	72.6%
% reporting established PCP	472

**Table 2:** Patients who called their PCP office prior to ED visit. Characteristics of patients who did and did not called primary care physician’s office prior to Emergency Department visit.

Characteristic	Called PCP before ED visit *n* (col %)	Did not call PCP before ED visit *n* (col %)	Odds ratio (95% confidence interval)
Gender			
Female	80 (22.5)	275 (77.5)	0.92 (0.64–1.3)
Male	73 (24.0)	232 (76.0)	
Income			
≤$20,000/year	98 (21.7)	353 (78.3)	1.2 (0.80–1.8)
>$20,000/year	39 (18.6)	170 (81.4)	
Education			
Less than high school	47 (23.7)	151 (76.3)	0.73 (0.50–1.1)
High school or higher	106 (30.0)	250 (70.2)	

**Notes.**

PCP Primary care physician ED Emergency Department

Patients with public and private insurance/self-pay were compared as to the frequency for which they called their PCPs office and whether they were sent to the Emergency department. We found that patients (91/329; 27.7%) with public insurance were significantly more likely to call their PCP prior to the ED visit than patients (7.5%; 25/330) with private or self-pay insurance (OR = 3.4; 2.9–7.4; *p* < 0.0001). Patients with public insurance (18.5% = 61/329) as compared with those who had private insurance (7.58%; 25/330) were also more frequently referred to the ED regardless of who they spoke to in the office (OR = 2.4; 1.4–4.0; *p* ≤ 0.001).

For patients who called their PCP office and believed that they were directed by phone to the ED (98 patients), the referral pattern is shown in Table 3. With respect to our primary outcome parameter, 31 out of 98 patients (31.63%; 23.2%–41.4%) who called their PCP’s office before coming to the ED reported that they had been directed there by a non-health care provider without physician input. An additional 11/98 (11.2%; 6.2–19.1%) only listened to a recorded message and felt the message was directing them to the ED. In our study 23 patients (14.8%; 10.0%–21.4%) who called their PCP had an appointment scheduled within 1–4 days but were still referred to come to the ED.

**Table 3:** Patients who called PCP office and were referred to ED with or without physician consultation (*N* = 98).

Source of referral to ED	Doctor consulted	Doctor not consulted
Non-Healthcare Personnel*	11/98 (11.2%)	31/98 (31.63%)
**Nurse**	14/98 (14.3%)	12/98 (12.3%)
**Phone Recording**	NA	11/98 (11.2%)

## Discussion

Investigators have previously examined the relationship between primary care offices and the ED from a variety of viewpoints, including non-emergent ED visits (New England Healthcare Institute, 2010; Massachusetts Division of Health Care Finance and Policy, 2004; Richardson & Hwang, 2001; Lowe et al., 1994; Coleman et al., 2001; Raven et al., 2013; Doran et al., 2013; Rocovich & Patel., 2012; American College of Emergency Physicians, 2011; Ravi et al., 2005). In a large national survey, Weinick, Billing and Thorpe estimated that 56% of all ED visits across the US are for non-emergent complaints that could be addressed in a primary care setting (New England Healthcare Institute, 2010). These findings were further validated in Massachusetts, where a state-wide universal health care system provides a large body of data to examine key public health issues within a captive population (Massachusetts Division of Health Care Finance and Policy, 2004). The Massachusetts Division of Health Care Finance and Policy examined 1 million ED visits over a one year period and determined that 46.5% of those patients could have been treated in a primary care office. Further, the investigators found that many of these ED visits occurred during normal business hours, not after hours when many physician offices are closed.

Our results should be viewed as preliminary, but certainly suggest directions for additional research to identify the impact of non-physician referral of patients on emergency department crowding. Including patients who believed they were referred to the ED by phone message, a total of 54 patients (8.1%; 6.3%–10.5) within our study group were directed to the ED after calling their PCP’s office but without physician input. This represents a relatively small percentage of patients that were referred to the ED without the consultation of a physician in the overall study population. However, this group represents over half of those patients that came to the ED after first calling their PCP, and felt like they were directed to the ED instead of the outpatient clinic. Within our own ED, an additional 5–10% of patients added to our daily census can significantly impact ED flow and overcrowding.

We surmise that our findings may reflect a symptom of a larger problem that has been identified in prior studies regarding the failure of communication between PCPs and the ED from a variety of perspectives (Rocovich & Patel., 2012; American College of Emergency Physicians, 2011; Ravi et al., 2005). A recent poll of ED physicians found that almost 97% had encountered patients referred to the ED by their PCP (Ravi et al., 2005). However, the ED physicians surveyed also noted that such referrals were rarely accompanied by a telephone conversation by the PCP and/or other communication (e.g., fax) to help explain the concerns and intent of the ED visit. Within other analyses, investigators also found that referrals to the ED were often unaccompanied by records/communication of the referring physicians evaluation of the patient (Rocovich & Patel., 2012; American College of Emergency Physicians, 2011).

We note that the solution to non-emergent type ED visits may not be as simple as providing better methods of communication between patients, PCPs, PCP office personnel, and emergency physicians. Phone triage to determine patient safety is a complex process, and, even when the patient is before qualified personnel, rapid assessment of acuity may not always be adequately accurate. Lowe, Bindman, Ulrich et al. performed a historical cohort study to establish the precision of guidelines utilized to identify patients safe for refusal of ED care. When two experienced nurses reviewed the charts of 106 patients who would have been refused care under such conditions, the investigators found that 35 (33%) had appropriate ED visits and four were hospitalized (38%) (Lowe et al., 1994).

## Limitations and Future Directions

This study has several limitations that warrant discussion. First, the study did not represent a true consecutive sample. Patients were enrolled consecutively during hours at which trained research associates were available. As the hours of the research associates varied throughout the hours of the day and week, we are hopeful that we surveyed a representative sample of our ED census including working and non-working patients. Second, as a novel study, our survey instrument has not been previously validated. It is possible that changes in the form of question could have afforded patients the opportunity to reflect more or less recall bias with respect to the phone call with their PCP office. In addition, the study was conducted during the winter and spring seasons which may have affected our results in view of the greater arrivals of seasonal illnesses when PCP offices are similarly overwhelmed with patients.

Our results may also only be pertinent in similar populations where the socioeconomic status is relatively low and most patients are under insured as well as underserved. There is a higher percentage of patients in this area that do not have primary care doctors, and, therefore, this may result in a greater influx of patients relying on the Emergency Department for medical care. Future studies should examine the question of primary care office referral source in suburban and other settings with demographics dissimilar from our center.

Likewise, additional investigation is warranted to further examine the question of features of patients more likely to be referred to the ED by the primary care office. We observed that patients with public insurance (18.5% = 61/329) as compared with those who had private insurance (7.58%; 25/330) were more frequently referred to the ED regardless of who they spoke to in the office (OR = 2.8; 1.7–4.5; *p* = < 0.0001). Whether or not this is due to economic incentives (i.e., higher reimbursement for a privately insured patient visit) or other features is unclear from our study. However, if similar studies from other settings confirmed this observation, it would suggest the need to, perhaps, regulate the availability of office visits in a manner similar to EMTALA/anti-dumping regulations. Physicians in non-hospital settings should be expected to participate in the care of patients from all socioeconomic classes rather than hospitals/EDs shouldering this burden alone.

## Conclusions

A relatively small percentage of patients were referred to the ED without the consultation of a physician in our overall population. However, of those that contacted their PCP’s office more than half were directed without a physician consult. Further study is warranted to assess the medical necessity/outcomes of these referrals and whether better methods of outpatient triage are needed to provide for appropriate utilization of emergency services.

## Supplemental Information

10.7717/peerj.1507/supp-1 Appendix S1 PCP Referral to the ED Data Collection Instrument PCP Referral to the ED Data Collection Instrument.

10.7717/peerj.1507/supp-2 Supplemental Information 1 PCP Referral the ED Data Set PCP Referral the ED Data Set

## Additional Information and Declarations

### Data Availability

The following information was supplied regarding data availability: The research in this article did not generate any raw data.
