# Supplementary material for: Are patients who call a primary care office referred to the emergency department by non-healthcare personnel without the input of a physician?
Source: PeerJ. 2016 Mar 28;4:e1507. doi: 10.7717/peerj.1507 (PMC4824903; doi:10.7717/peerj.1507)
Supplement: Appendix S1 — PCP Referral to the ED Data Collection Instrument. [file peerj-04-1507-s001.docx]

Your participation in this survey is completely voluntary and will have no effect on your relationship or care with CHRISTUS Spohn Hospital Corpus Christi--Memorial as a patient. There will be no financial benefits for participating in this survey. Please know that we will do everything we can to protect your privacy.

**Do you agree to voluntarily be a subject of this research study?** **YES________ No_______**

**Please circle the information below about yourself:**

1. Sex: Male^0^ Female^1^

2. Age in years: ___________ **(If over 89 check here only)**

3. Race: Caucasian/White^1^ Hispanic^2^ Native American^3^ Asian^4^ Non-Hispanic/Black^5^ Other^6^

4. What is your household’s yearly annual income (estimate)?

$0-$20,000^1^ $20,001-$40,000^2^ $40,001-$60,000^3^ $60,001-$80,000^4^ $80,001 or more ^5^

5. What is the highest level of education you completed in school?

Less than High School^1^ High School Graduate^2^ Some College^3^ College Graduate^4^ Any Post Graduate Work^5^

6. What type of health insurance do you have?

Private insurance^1^ Nueces Aid (“Clinic Card”) ^2^ Medicaid^3^ Medicare^4^ Self-Insured^5^ None^6^

7. What brought you to the ER today?

Pain^1^ Infection^2^ Injury^3^ Vomiting/Diarrhea^4^ Out of Medications^5^ Other^6^______________

8. Did you call your primary care physician or regular doctor today?

Yes^1^ No^0^  I do not have a doctor^3^

**If you answered “No” or “I do not have a doctor” please turn in your survey now.**

9. Who did you speak to when you called your primary care physician or regular doctor?

Doctor^1^ PA/NP^2^ Receptionist^3^  Nurse^4^ Recorded Message^5^

10. Did the person you spoke with on the phone consult with your doctor?

Yes^1^ No^0^

11. What did the person you spoke with or recording ask you to do?

Go to the ER^1^ Come in today^3^ Schedule an appointment with your physician^2^

12. If you scheduled an appointment, when is it?

No appointment was scheduled^1^  1-2 days^2^ 2-4 days^3^ more than 1 week^4^ more than 1 month^5^
